# Therapeutic effect of dual CAR-T targeting PDL1 and MUC16 antigens on ovarian cancer cells in mice

**DOI:** 10.1186/s12885-020-07180-x

**Published:** 2020-07-20

**Authors:** Tong Li, Jiandong Wang

**Affiliations:** Department of Gynecologic Oncology, Beijing Obstetrics and Gynecology Hospital, Capital Medical University, Beijing, 100006 China

**Keywords:** Chimeric antigen receptor T cell, Mucin 16, Programmed cell death-ligand 1, Ovarian cancer

## Abstract

**Background:**

More favorable treatment against epithelial ovarian cancer (EOC) is urgently needed because of its insidious nature at an early stage and a low rate of five-year survival. The current primary treatment, extensive surgery combined with chemotherapy, exhibits limited benefits for improving prognosis. Chimeric antigen receptor T (CAR-T) cell technology as novel immunotherapy has made breakthrough progress in the treatment of hematologic malignancies, and there were also benefits shown in a partial solid tumor in previous research. Therefore, CAR-T cell technology may be a promising candidate as an immunotherapeutic tool against EOC. However, there are some weaknesses in targeting one antigen from the previous preclinical assay, such as on-target off-tumor cytotoxicity. The dual-target CAR-T cell may be a better choice.

**Methods:**

We constructed tandem PD1-antiMUC16 dual-CAR, PD1 single-CAR, and anti-MUC16 single-CAR fragments by PCR and genetic engineering, followed by preparing CAR-T cells via lentiviral infection. The expression of CAR molecules on single and dual CAR-T cells was detected by flow cytometry. The killing capacity and activation of CAR-T cells were measured by cytotoxic assays and cytokines release assays in vitro. The therapeutic capacity of CAR-T cells was assessed by tumor-bearing mice model assay in vivo.

**Results:**

We successfully constructed CARs lentiviral expression vectors and obtained single and dual CAR-T cells. CAR-T cells demonstrated robust killing capacity against OVCAR-3 cells in vitro. Meanwhile, CAR-T cells released plenty of cytokines such as interleukin-2(IL-2), interferon-γ (IFN-γ) and tumor necrosis factor-α(TNF-α). CAR-T cells showed a therapeutic benefit against OVCAR-3 tumor-bearing mice and significantly prolonged the survival time. Dual CAR-T cells were shown to be two to four times more efficacious than single CAR-T cells in terms of survival time.

**Conclusion:**

Although exhibiting a similar ability as single CAR-T cells against OVCAR-3 cells in vitro, dual CAR-T cells demonstrated enhanced killing capacity against OVCAR-3 cells as compared to single CAR-T cells in vivo and significantly prolonged the survival time of tumor-bearing mice. PD1-antiMUC16 CAR-T cells showed more potent antitumor activity than single CAR-T cells in vivo. The present experimental data may support further research work that will have the potential to lead to clinical studies.

## Background

Epithelial ovarian cancer (EOC) represents approximately 90% in Ovarian cancer (OC), which is the fifth most common tumor in female malignancies [1, 2]. EOC is classified as a serous, endometrioid, mucinous, clear cell and unspecified type in the tumor cell histology [3]. More than 50% of serous carcinoma is the primary type of EOC [4], and it is diagnosed at stage III (51%) or stage IV (29%) due to the absence of specific early symptoms [3]. Due to inadequate screening and detection methods at early stage, more effective and less recrudescent therapies are urgently needed. The current primary treatment of EOC is extensive surgery combined with platinum-based or taxane-based chemotherapy, however, there are limited benefits for improving prognosis [2–4].

CAR-T cell therapy as one of the representative adoptive immunotherapies, has made unprecedented progress in the treatment of hematologic malignancies. The US Food and Drug Administration (FDA) has approved CD19 CAR-T products for acute lymphoblastic leukemia and diffuse-large B cell lymphoma [5]. However, because of the deficiency of tumor-specific targets and physiologic barrier, it is challenging for the patients with solid tumors to receive benefits [6].

Some researchers engineered multiple CAR-T cells on OC in numerous studies and demonstrated desirable outcomes. For example, the NKG2D-CAR-T cell can specifically recognize and kill the OC cells expressing NKG2DL antigen [7]. CAR-T cells can recognize and combine with the tumor cells expressing specific antigen via extracellular scFv fragment [8]. After recognizing the target cells, CAR-T cells release cytokines such as IL-2, IL-6, TNF-α, and IFN-γ to activate T cells and stimulate NK cells promoting the secretion of various factors that starts a series of killing effect [9]. However, most CAR-T cell has one specific CAR molecule that targets one antigen of the tumor cells, which may cause on-target off-tumor toxicity, difficulty in homing, absence of sustaining T cell effect and cytokine release syndrome (CRS) in vivo [10, 11]. In addition, single CAR-T cannot improve the tumor microenvironment. The immune escape caused by influence of the tumor microenvironment cannot be avoided [12]. Considering the overall lethal effect and deficiency of single-target CAR-T technology in carcinomas, we hypothesized a CAR-T with higher specificity, i.e. dual-target CAR-T, would address the deficiency while exhibit an enhanced lethal effect on EOC. In structuring valiant dual-target CAR-T, selecting specific antigens as targets are very crucial.

Mucin 16 (MUC16), as the glycoprotein with the highest massive molecule weight in the mucin family, is a critical biomolecule to maintain the intracellular balance and protect the epithelium [13]. It is expressed in a variety of tumor cells and involved in the proliferation and metastasis of tumor cells. Studies have shown that 80% EOC express MUC16, and its extracellular segment is cut and released in the peripheral blood to be a well-known tumor marker, namely CA125 [14]. Therefore, we believe that MUC16 is an ideal antigenic target for CAR molecules.

Programmed cell death-1(PD1) is an immunosuppressive molecule widely expressed on the surface of activated T cells, B cells, antigen-presenting cells, and macrophages. It belongs to the CD28/cytotoxic T lymphocyte-associated antigen-4(CTLA-4) family [15]. PD-1 and its ligand PDL1 constitute the PD1/PDL1 signaling pathway, which plays an inhibitory role in T cell immunity. Current research suggests that T cells can secrete cytokines such as IL-10 and IFN-γ to induce generation of CTLA ligand, such as PD1 expressing on OC cells. At the same time, PD1 induces expression and combines with inhibitory receptors on the surface of T cells, therefore reducing the anti-activity of effector T cells and guiding T cell reposition or causing T cell failure to achieve immune escape [16–20]. In the experiment of melanoma-bearing mice, it was found that the up-regulated expression of PDL1 in the tumor microenvironment led to the suppression of anti-tumor immune escape on T cells. After intraperitoneal injection of the PD1 antibody to block the PD1 pathway, T cell significantly increased infiltration [21–23]. There is also a study that shows the five-year survival rate of patients with low expression of PDL1 is significantly higher than that of patients with high expression of PDL1 [22, 24, 25]. Based on the above research of PD1, we surmise that PDL1 would be another ideal target.

In recent years, more researches have been done on PD1 in various solid tumors, such as breast cancer and prostate cancer. There are evidences that PD1 positively contributes to CAR-T cells function [26, 27]. Liu X et al. demonstrated that when PD1 co-expressed with anti-mesothelin on CAR-T cells against multiple solid tumor cells, the PD1 chimera enhanced the effector activity of CAR-T cells in mice model [28]. Therefore, it is reasonable to speculate that the combination of PD1 and anti-MUC16 is one of the best combinations of CAR-T against EOC.

In this study, we developed a novel tandem-specific CAR-T cell that targets MUC16 and PDL1 antigens. We investigated whether the extracellular domain of PD1-antiMUC16 CAR-T can effectively recognize the targeted antigens, kill tumor cells, and further release cytokines and prolong the survival time of tumor-bearing mice.

## Methods

### Cell lines

Lenti-X 293 T cell line was purchased from Clontech (Cat#632180, California, United States) in August 2018, and the test report was provided by Clontech (California, United States). OVCAR-3 cell line was purchased from FuHeng Cell Center (Cat#FH0726, Shanghai, China) in February 2016. It was tested by STR Authenticated provided by FuHeng Cell Center, and the test report showed a complete match with the NIH: OVCAR-3 (ATCC HTB-161). Umbilical blood mononuclear cell (UBMC) was obtained from healthy donors in Beijing Obstetrics and Gynecology Hospital in March 2019. UBMC was tested for the positive rate of CD3 on the cell surface by flow cytometry after being activated. All cell lines were validated monthly to be mycoplasma free by PCR. Lenti-X 293 T cells were used to construct lentiviral expression vectors and were cultured in high glucose DMEM medium (Hyclone, Logan, United States) containing 5% fetal bovine serum (FBS, Hyclone, Logan, United States) and 1% penicillin-streptomycin solution (Hyclone, Logan, United States). OVCAR-3 cells were marked as OVCAR3-luc cells by luciferase, culturing in RPMI-1640 medium (Gibco, California, United States) supplemented with 10% FBS, 1% penicillin-streptomycin solution, and 0.1% insulin (Gibco, California, United States). After being activated by anti-CD3/CD28 magnetic beads (Novoprotein, Shanghai, China), UBMC was cultured in the GT-T551 H3 medium (TaKaRa, Osaka, Japan) containing 5%FBS and 40 IU/ml IL-2 (Novoprotein, Shanghai, China). All cells were cultured in an incubator (ESCO, Portland, United States) at 37 °C and 5% CO_2_. This study was approved by the Medical Ethics Committee, Beijing Obstetrics and Gynecology Hospital, Capital Medical University (2018-KY-026-01).

### Construction of CAR molecule

After designing the sequences, primers and templates were synthesized by Sangon Biotech. According to the PCR principle, the single chain antibody fragments (scFv) of PD1, anti-MUC16 and PD1-antiMUC16 CAR were obtained. The main structures of PD1 and anti-MUC16 are PD-1etco and 4H11-VH-(Gly4Ser)3-4H11-VL. The primary structure of PD1-antiMUC16 is tandem of PD1 and anti-MUC16. The three scFv fragments were cloned into the pLVX-EFlα-IRES-mCherry plasmid (Clontech, California, United States) through EcoR I and Mlu I cloning sites and named PD1-antiMUC16 CAR, anti-MUC16 CAR, and PD1 CAR, respectively. The plasmid has been genetically engineered to be a second-generation CAR containing CD8a hinge region, CD8 transmembrane region, 4-1BB co-stimulation domain, and CD3ζ domains. The plasmids were amplified in bacterial solution, and the positive samples were selected by agarose gel electrophoresis and verified via sequencing analysis.

### Lentivirus packaging

Set the experiment groups (pLV-PD1-anti-MUC16, pLV-anti-MUC16, and pLV-PD1) and Control group (control T). In each group, 13.7 μg plasmid was taken and mixed with packaging plasmid containing 3.43 μg pMD2.G, 3.43 μg pMDLg/pRRE, 3.43 μg pRSV-Rev (Addgene, Massachusetts, United States) to make DNA-mix. 7 × 10^6^ cells Lenti-X 293 T were added into the DNA-mix and the same volume of polyethylenimine (PE1, Polyscience, Pennsylvania, United States), then cultured in the incubator. Fresh virus packaging medium containing Opti-MEM (Gibco, California, United States), 5% FBS, 1% L-glutamine (Gibco, California, United States), 1% sodium pyruvate (Gibco, California, United States), and 0.2% penicillin-streptomycin solution was supplemented after 6 h of culturing. After 24 h, we collected supernatant and obtained virus concentrate (210 μL), from which 6 μL was taken to infect 293 T cells again for virus titer detection. The rest of the virus concentrate was stored in a 4 °C refrigerator for preparation of CAR-T cells. After 48 h of infection, the infected cells were placed into 12-well plates (Corning, New York, United States). 5 μL Percp-cy5.5 antihuman PD-1 antibody (BD, New jersey, United States), and 10 μL FITC-Protein L antibody (ACRO, Delaware, United States) were added to each well, and incubated in the dark for 30 min. The flow cytometry (NovoCyte Advanteon, ACEA, Hangzhou, China) was used to detect the positive rate of PD1 and anti-MUC16 in Lenti-X 293 T cells. The viral titer was calculated according to the following formula:

1 $$ \mathrm{Viral}\ \mathrm{Titer}\left(\mathrm{TU}/\mathrm{ml}\right)=\frac{\left(\mathrm{Number}\ \mathrm{of}\ \mathrm{Infected}\ \mathrm{Cells}\times \mathrm{Positive}\ \mathrm{Rate}\right)}{\mathrm{Viral}\ \mathrm{Volume}\left(\mathrm{ul}\right)}\times 1000 $$

### T cell transduction

Set the experiment groups (pLV-PD1-anti-MUC16, pLV-anti-MUC16, and pLV-PD1) and Control group (control T). After thawing, 2.5 × 10^6^ UBMC were added into phosphate buffer saline (PBS, Hyclone, Logan, United States) with a volume of 10 times, which was then centrifuged at 1000 rpm for 15 min (BT-320C, Baiyang, Beijing, China). Then an appropriate T cell complete medium and 25 μL magnetic beads were added. The mixture was cultured in the incubator for 48 h. Lentiviral supernatants were collected, of which 200 μL per well was added into the 12-well plate coated with 200 μg NovoNectin (Novoprotein, Shanghai, China) overnight. Meanwhile, control T well was added with 200 μL GT-T551 H3 medium. All wells were added with 800 μL UBMC (2.5 × 10^5^cells/well) and cultured in an incubator. After culturing for 6 h, 2 mL of T cells complete growth medium (GT-T551 H3 + 5%FBS + 40 IU/ml IL-2) was added and T cells were re-infected for the following day. After the second infection for 96 h, 1 × 10^6^ cells was taken out from each well, washed twice with PBS, removed of the magnetic beads, and stained with 5 μL Percp-cy5.5 antihuman PD-1 antibody and 10 μL FITC-Protein L antibody for 30 min in the dark. Then it is followed by washing with PBS twice and detection for the positive rate of CAR structure by the flow cytometry. Finally, when CAR-T cells were successfully prepared, the positive ratio of the other two CAR-T cells was adjusted according to the lowest positive ratio of three CAR molecules by increasing the number of control T cells.

### Preparation of target cells

After thawing, OVCAR3-luc cells were cultured in RPML1640 medium containing 20% FBS, 1% glutamine, 1% sodium pyruvate, 1% penicillin and streptomycin,0.01 mg/mL insulin. It was seeded in T25 bottle with a density of 5 × 10^5^/4 cm^2^ in an incubator (37 °C, 5% CO_2_). Both MUC16 plasmid and PDL1 plasmid (Juventas, Tianjin, China) were mixed with the PEI-DNA mix to complete lentiviral packaging by PEI transfection, and were harvested for MUC16 lentiviral expression vector, PDL1 lentiviral expression vector, and MUC16-PDL1 lentiviral expression vector. The lentiviral concentrate was harvested at 24 h and 48 h, followed by infecting OVCAR3-luc cells. Flow cytometry measured the positive rate of MUC16 and PDL1 on the OVCAR3-luc cell surface after 96 h of re-infection.

### Flow Cytometry

One million CAR-T cells were stained with 5 μL Percp-cy5.5 antihuman PD-1 antibody and 10 μL FITC-Protein L antibody for an antigen-antibody binding reaction. After 30 min of dark incubation, dissociative antibodies uncombined with antigens were washed by PBS. CAR molecule expression was measured for fluorescence intensity by flow cytometry. This method was used to detect viral titer and transfection rate of T cell. The cytotoxicity of CAR-T cells was evaluated via detecting the luciferase expression of tumor cells after stained with 70 μL Steady-Glo. Simultaneously, the transfection efficiency of tumor cells was evaluated via detecting green fluorescent protein (GFP).

### Enzyme-linked Immunosorbent assay (ELISA)

The human IFN-gamma ELISA kit, IL-2 ELISA kit, and TNF-alpha ELISA kit (all kits, Dakewe, Beijing, China) were used to measure the concentrations of IFN-γ and IL-2, TNF-α, respectively. According to the instructions of the ELISA kit, three samples were processed, and the standard curve was prepared. Then the fluorescence value was measured by the enzyme-labeled instrument (TECAN, Mannedorf, Switzerland). The cytokines quantity was then calculated.

### Cytotoxicity assay and cytokine release assay

Target cells: OVCAR3-luc cells, OVCAR3-PDL1-luc cells, OVCAR3-MUC16-luc cells, and OVCAR3-MUC16-PDL1-luc cells; Effector cells: PD1-antiMUC16 CAR-T cells, antiMUC16 CAR-T cells, PD-1 CAR-T cells, control T cells (negative group). Target cells was adjusted for a density of 2 × 10^5^cells/mL by GT-T551 H3, and seeded into the black flat 96-well U-bottomed plate (50 µL/well). Effector cells were adjusted for a density of 3.2 × 10^6^cells/mL by GT-T551 H3, and seeded into the same 96-well plate (50 μL/well) at effector to target(E/T) ratios of 1:1, 4:1, 8:1 and 16:1, respectively. Simultaneously, target cells (50 μL/well) were cultured alone and with 50 μL GT-T551 H3 medium. The plate was put in the incubator at 37 °C for 4 h. Afterwards, all cells were stained with 70 μL Steady-Glo (Promega, Wisconsin, United States) per well for 20 min in the dark. The fluorescence value was detected by flow cytometry and the killing rate of various CAR-T cells on tumor cells was calculated according to the following formula:

2 $$ \mathrm{Killing}\ \mathrm{Rate}\%=\frac{\left(\mathrm{Fluorescencemax}\ \mathrm{of}\ \mathrm{target}\ \mathrm{cells}-\mathrm{Fluorescence}\ \mathrm{of}\ \mathrm{experimental}\ \mathrm{well}\right)}{\mathrm{Fluorescencemax}\ \mathrm{of}\ \mathrm{target}\ \mathrm{cells}}\times 100\% $$

The CAR-T cells were co-cultured with target cells, at 1:1 ratio(1 × 10^4^ T cells and 1 × 10^4^ target cells) in a V-bottomed 96-well plate for 48 h in the incubator, followed by harvesting supernatant through centrifugation (2500 rpm, 5 min), and detection for the release of IFN-γ, IL-2, TNF-α, respectively by ELISA kit.

### Xenograft mice models for in vivo treatment

All animal studies were approved by the Medical Ethics Committee, Beijing Obstetrics and Gynecology Hospital, Capital Medical University (2018-KY-026-01). In-house bred NPG mice (NOD.Cg-PrkdcscidIl2rgtm1Vst/Vst) were obtained from Beijing Vitalstar Biotechnology Co., Ltd. Twenty healthy NPG mice (females, 35–41 days old, 18-21 g in weight) were raised in specific pathogen-free (SPF) conditions and fed with autoclaved food and water. For the xenograft models, NPG mice were intraperitoneally injected with 5 × 10^5^ OVCAR3-MUC16-GFP-PDL1-luc cells and 50 μL Matrigel (Corning, New York, United States). After 48 h, mice were intraperitoneally injected with 100 μL D-Luciferin and Postassium Salt (Sciencelight, Shanghai, China) for 6 min before being put in an isoflurane-oxygen mixture gas anesthetic box containing 3% isoflurane (RWD, Shenzhen, China) for 2 min. The tumor burden was then measured by IVIS Spectrum and analyzed by Living Image, version 4.3, software (Perkin Elmer). The study subjects were distributed randomly into four groups (*n* = 5 for each group) on day 0, and each group was intraperitoneally injected with CAR-T cells (1 × 10^6^cells per mouse). Imaging was performed on days 7, 14, 21 and 28 to monitor the tumor changes. Mice were euthanized by carbon dioxide asphyxiation when tumour volume exceeded 2000 mm^3^, when the weight loss over 20%, or when they lost their ability to eat autonomously. The mice were placed in a transparent box that released pure carbon dioxide after conformed to the above criteria. When mice were observed to faint, carbon dioxide continued to be released for 2 min for euthanization. The survival time of each mouse was recorded and the survival curve by GraphPad Prism 8.3.0 software drawn.

### Statistical analysis

Statistical analysis was performed using SPSS 23.0 software. Data are shown as mean ± 1 standard deviation (SD). For the in vitro killing assay, the significance of different groups was determined using nonparametric tests. For the in vivo assay, Student’s t-test was used to distinguish the difference between groups. The value of *P* < 0.05 was considered significant. The mice survival curve was drawn using GraphPad Prism 8.3.0 software.

## Results

### Construction of dual-target CAR-T cells by Lentiviral vector transduction

**Fig. 1:**
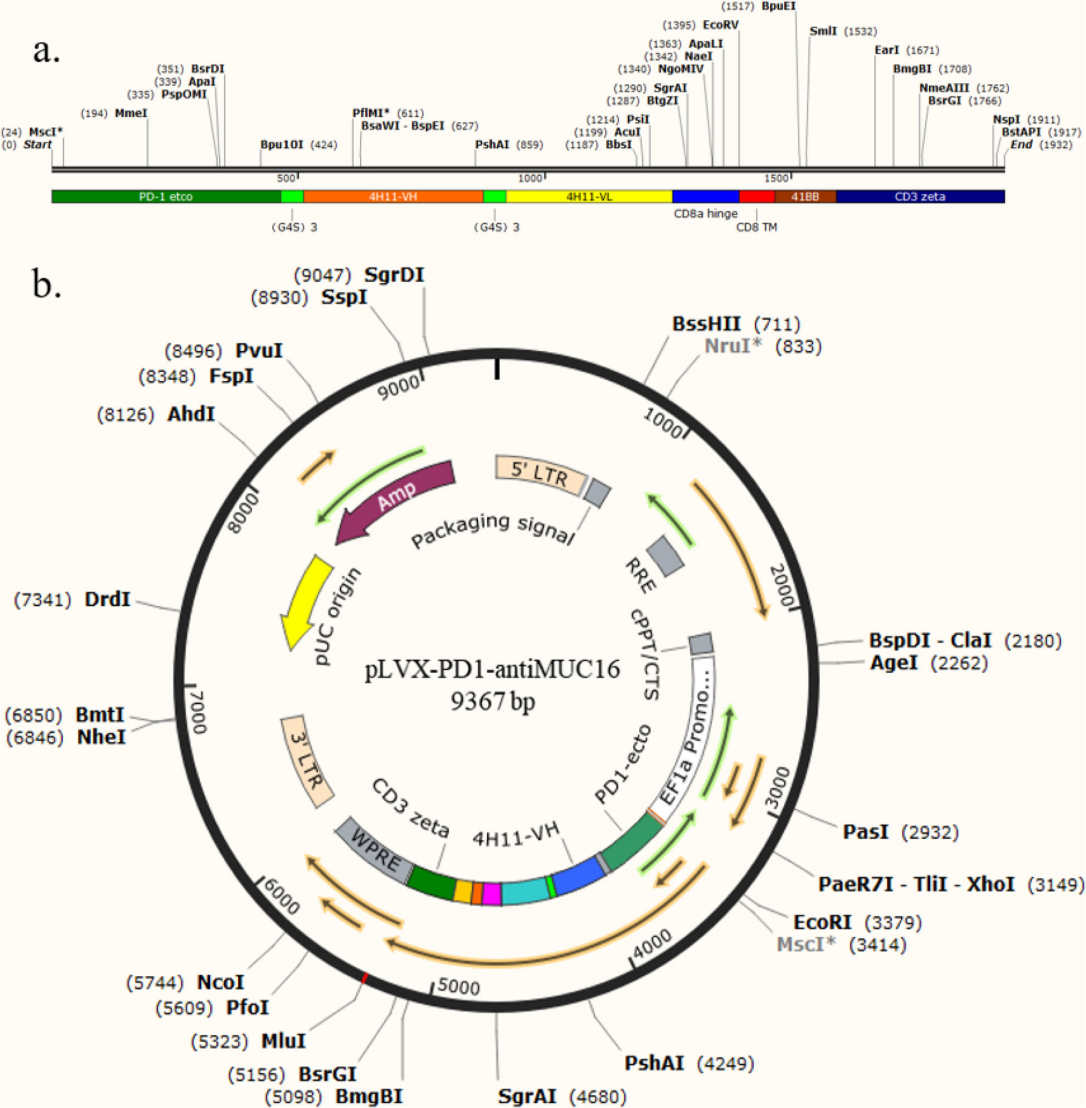
Construction of dual CAR molecule and PD1-antiMUC16 plasmid. **a** Schematic diagram of dual-target CAR molecule transgene. PD1-antiMUC16 CAR structure shows the five parts, extracellular PD1-antiMUC16 scFv, a CD8a hinge, a transmembrane region, 4-1BB co-stimulation domain, and CD3ζdomain, among the structure anti-MUC16 that consist of 4H11-VH and 4H11-VL. **b** Schematic diagram of dual-target CAR expression vector. PD1-antiMUC16 plasmid structure shows base sequence sites and the sites of enzyme digestion

**Fig. 2:**
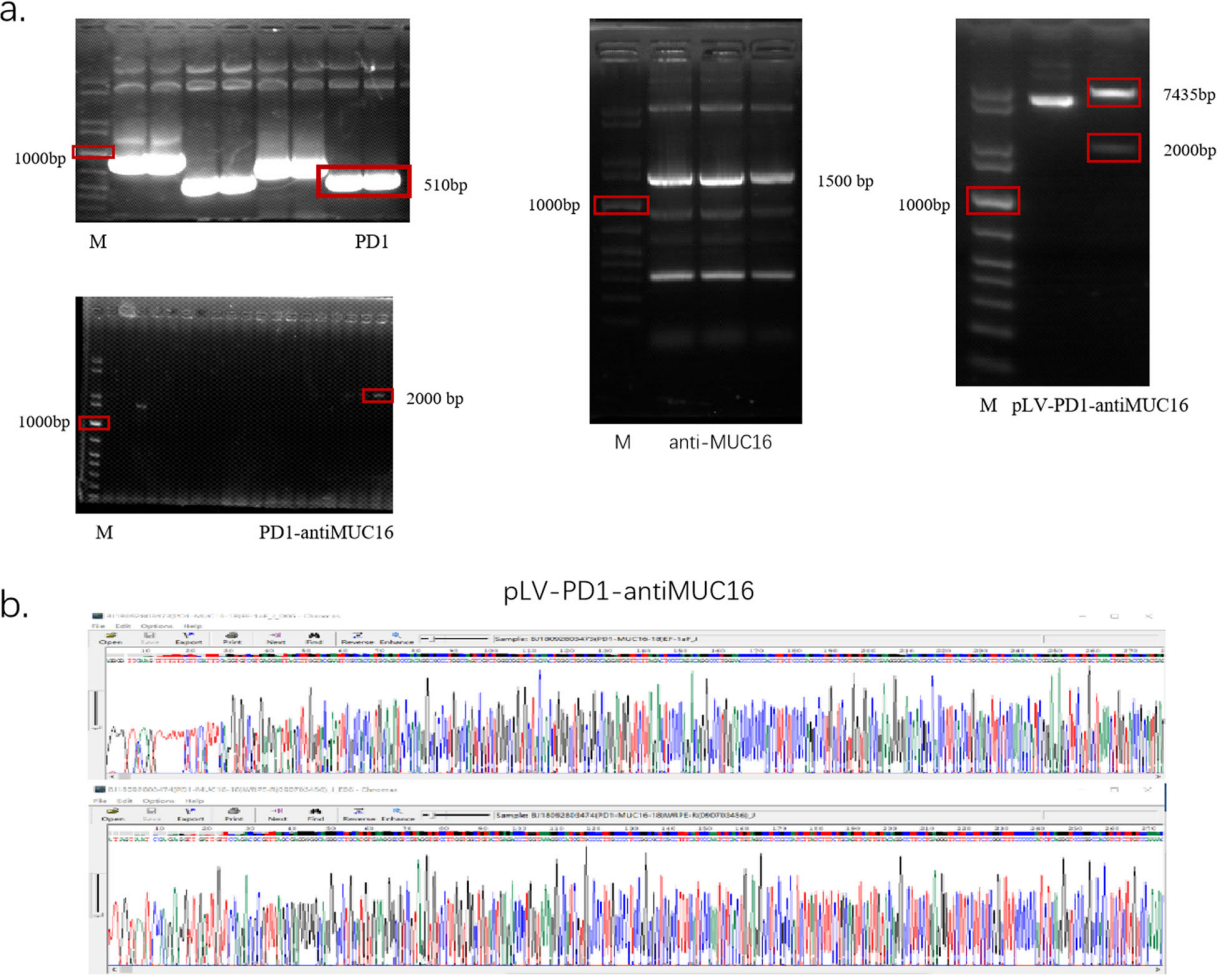
Detection results of PD1-antiMUC16 CAR by agarose gel electrophoresis and sequencing. **a** CARs detected by agarose gel electrophoresis, M represents 1Kb marker, the right bands of PD1, anti-MUC16, and PD1-antiMUC16 CARs were circled in red. **b** The base sequence of the PD1-antiMUC16 CAR structure is quite correct. The full-length gels are presented in Additional file 1: Figure S1

**Fig. 3:**
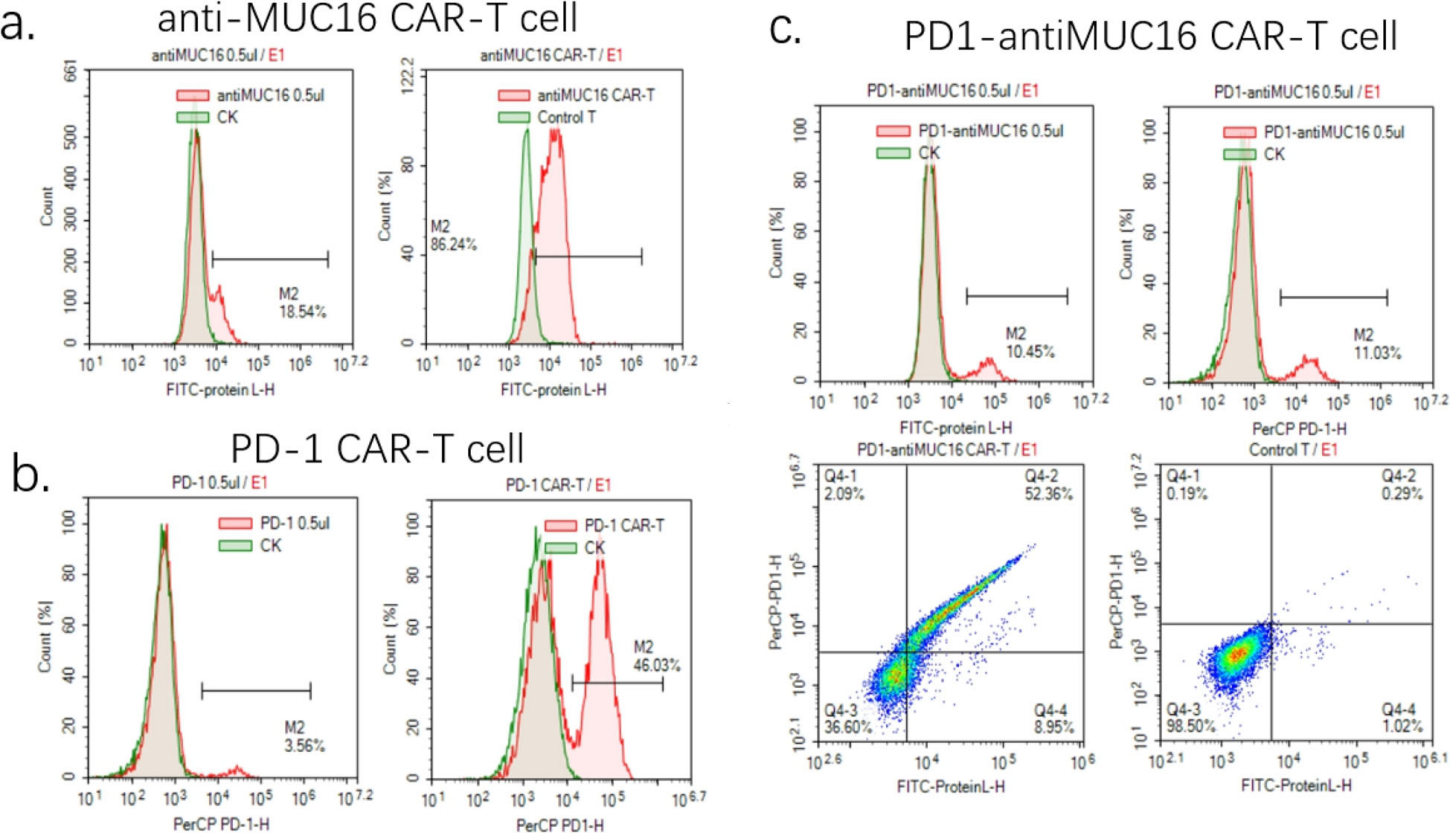
Detection results of the positive rate of single and dual CARs on T cells. **a** The testing results of viral titer and infection rate of anti-MUC16 CAR-T cell. Anti-MUC16 CAR-T cells stained with 10 μL FITC-Protein L antibody for 30 min in the dark, followed by detecting via flow cytometry. The green peak is the control group, and the red peak is the experimental group. **b** The detecting results of viral titer and infection rate of PD1 CAR-T cell. PD1 CAR-T cells stained with 5 μL Percp-cy5.5 antihuman PD-1 antibody for 30 min in the dark, followed by detecting via flow cytometry. **c** The testing results of viral titer and infection rate of PD1-antiMUC16 CAR-T cell. Control T showed both negative results stained with two antibodies. However, PD1-antiMUC16 CAR-T showed both positive results with two antibodies

According to the above protocol, we designed the PD1-antiMUC16 CAR molecule structure, which comprised PD1-antiMUC16 or PD1 or anti-MUC16 extracellular scFv fragment, a hinge region, a transmembrane domain, followed by intracellular 4-1BB co-stimulation domain and CD3ζ domain. In addition, the scFv fragment contained three parts, i.e., PD1 ecto, 4H11-VH (heavy chain) and 4H11-VL (light chain). These three parts were connected by the linker peptide (Gly4Ser)3 that constructed the 2000 bp dual-target CAR molecule (Fig. 1a). Through gene recombination, CAR molecule sequences combined with the 9367 bp lentiviral vector, which were digested by two enzymes (EcoR I and Mlu I) (Fig. 1b). Via testing by agarose gel electrophoresis, PD1, anti-MUC16, PD1-antiMUC16, and plasmid skeleton fragment bands were observed at 510 bp,1500 bp,2000 bp, and 7435 bp, respectively (Fig. 2a), and original gel electrophoresis was in Additional file 1 (Figure S1). Meanwhile, the sequence of the positive samples was analyzed and verified to be entirely consistent with the designed one (Fig. 2b). The plasmids were transferred into Lenti-X 293 T cells to package lentivirus by polyetherimide (PEI) transfection. Anti-MUC16 and PD1 antigen were combined with 10 μL FITC-Protein L antibody and 5 μL Percp-cy5.5 antihuman PD-1 antibody, respectively, followed by detection of positive rate of CARs by flow cytometry. Thereout, we harvested the favorable rates of PD1-antiMUC16 CAR, PD1 CAR, and antiMUC16 CAR on the surface of Lenti-X 293 T cells, which were 10.45,3.56, and 18.54%, respectively (Fig. 3). Three viral titers were obtained respectively: 6.27 × 10^7^TU/mL, 2.14 × 10^7^TU/mL, and 1.11 × 10^8^TU/mL according to the Formula (1). Utilizing the same detection methods, the infection rates of PD1-antiMUC16 CAR-T cells, PD1 CAR-T cells, and anti-MUC16 CAR-T cells were obtained (52.36, 46.03, and 86.24%, respectively) (Fig. 3). It indicated that CAR-T cells with single- and dual-targets were successfully constructed.

### Overexpressing MUC16 and PDL1 antigens of target cells

**Fig. 4:**
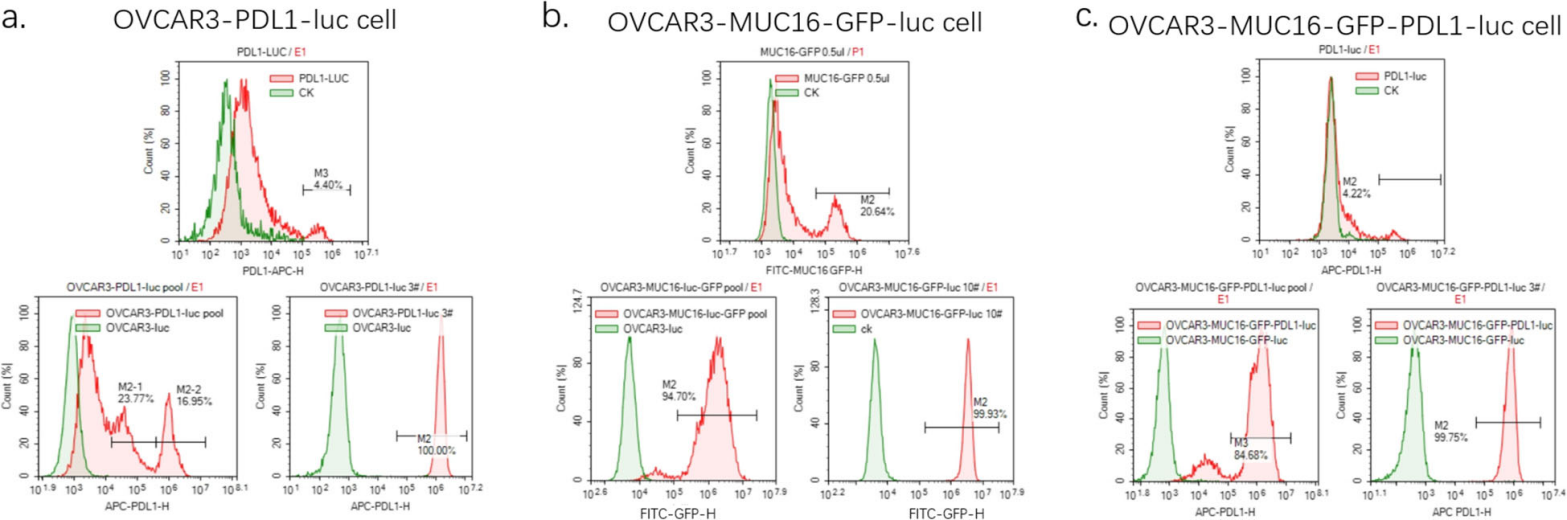
Detection results of target cells overexpressing PDL1 and(or) MUC16. **a** Detection results of PDL1 antigen on OVCAR3-PDL1-luc cells. OVCAR3-PDL1-luc cells stained with 70ul Steady-Glo for 20 min in the dark. **b** Detection results of MUC16 antigen on OVCAR3-MUC16-GFP-luc cells. GFP is used to mark the MUC16. **c** Detection results of MUC16 and PDL1 antigens on OVCAR3-MUC16-GFP-PDL1-luc cells. OVCAR3-MUC16-GFP-PDL1-luc cells stained with 70ul Steady-Glo as the above methods. Control groups are the green peak; Experimental groups are the red peak. The top pictures show the titer test results, and the bottom two show the cell pools and the monoclonal samples

Based on the above principles and methods with PCR, genic recombination, lentiviral vector transduction, we acquired the 4.40% positive rate of PDL1 molecule on the surface of the Lenti-X 293 T cell after combining with 5 μL Percp-cy5.5 antihuman PD-1 antibody for 30 min in the dark (Fig. 4a). The lentiviral titer was 2.64 × 10^7^TU/mL, according to the Formula (1). The infection rate of the PDL1 structure on OVCAR3-luc cell was achieved at 40.72% (Fig. 4a). The population that consists of OVCAR3-PDL1-luc cells and unstructured cells was named a pool. The positive samples were cultured and proliferated, followed by purification to 100% (No.3 monoclonal sample) (Fig. 4a). Meanwhile, the lentiviral titer of MUC16 in the MUC16 group was 1.24 × 10^8^TU/mL. MUC16-GFP in the OVCAR3-MUC16-GFP-luc pool was selected based on the expression GFP with the MUC16 monoclonal sample purified to 99.93% (Fig. 4b). The MUC16-GFP positive rate was 94.70%. Moreover, we created MUC16-PDL1 antigen via structuring the PDL1 directly on the monoclonal sample of MUC16. The lentiviral titer of PDL1 in MUC16-PDL1 group was 2.52 × 10^7^TU/mL, and the positive rate of PDL1 on OVCAR3-MCU16-GFP-luc pool was 84.68% with the PDL1 depurated to 99.75% (Fig. 4c). OVCAR3-luc cell lines overexpressing MUC16 and PDL1 antigens were successfully constructed, and the positive rate of tumor cell surface antigen was over 99%.

### Functional activity of CAR-T cells in vitro

**Fig. 5:**
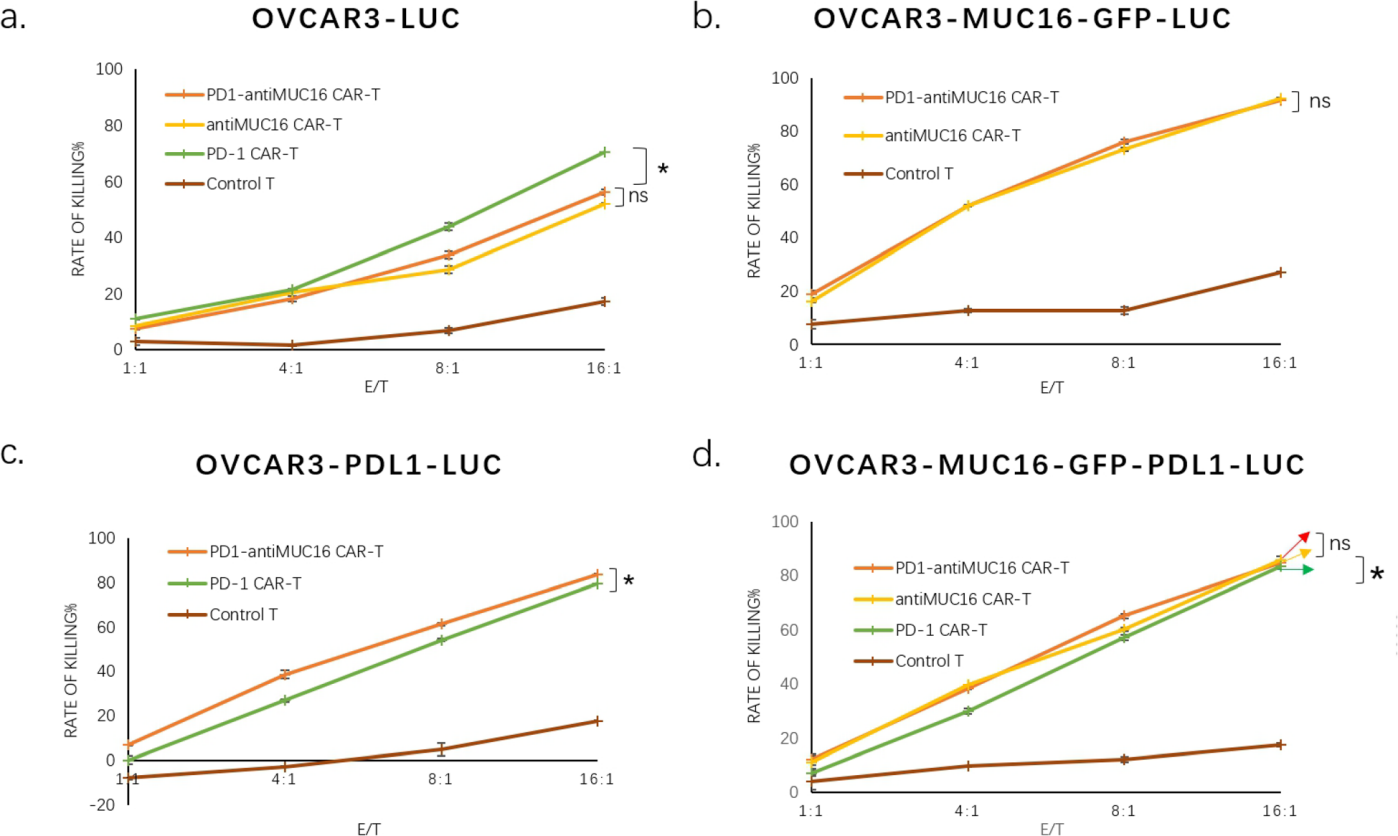
The antitumor activity results in single and dual CAR-T cells against various target cells. There are four kinds of tumor cells expressing PDL1 and(or) MUC16 antigen or not, and four different T cells expressing PDL1 and(or) antiMUC16 or not. The CAR-T cells co-cultured for 4 h with target cells (1 × 10^4^) at E/T of 1:1,4:1,8:1, and 16:1 in a total volume of 100ul, after that stained with 70 μL Steady-Glo for 20 min and detected by flow cytometry. Data show the mean ± SD, and the results were analyzed with the nonparametric test. Error bars represent the SD. ns: *P* > 0.05, *: *p* < 0.05

To ascertain the cytotoxicity of CAR-T cells against MUC16 or PDL1 positive cancer cells in vitro, we co-cultured CAR-T cells and cancer cells (1 × 10^4^cells/well) at 1:1, 4:1,8:1, 16:1 ratio. We performed killing assay of PD1-antiMUC16, PD1, and anti-MUC16 CAR-T cells on various target cells for 4 h. The assay indicated that the dual-target CAR-T cells exhibited more potent cytotoxicity than control T cells against any target cell (*P* < 0.05), and the capacity was enhanced with the increase of E/T ratio (P < 0.05). The killing rates of PD1-antiMUC16 CAR-T cell on OVCAR3-MUC16-GFP-PDL1-luc cells were 12.03 ± 1.98%, 38.29 ± 0.13%, 65.16 ± 0.95%, and 84.96 ± 0.53% at E/T ratios of 1:1, 4:1, 8:1, and 16:1, respectively. Comparatively, the killing rates of the control T cells was 3.90 ± 2.76%, 9.74 ± 0.13%, 12.20 ± 0.95%, and 17.56 ± 0.75%, respectively. Meanwhile, single-target CAR-T cells, PD1 and anti-MUC16 CAR-T remained almost the same cytotoxicity efficacy with dual CAR-T cell (Fig. 5).

**Fig. 6:**
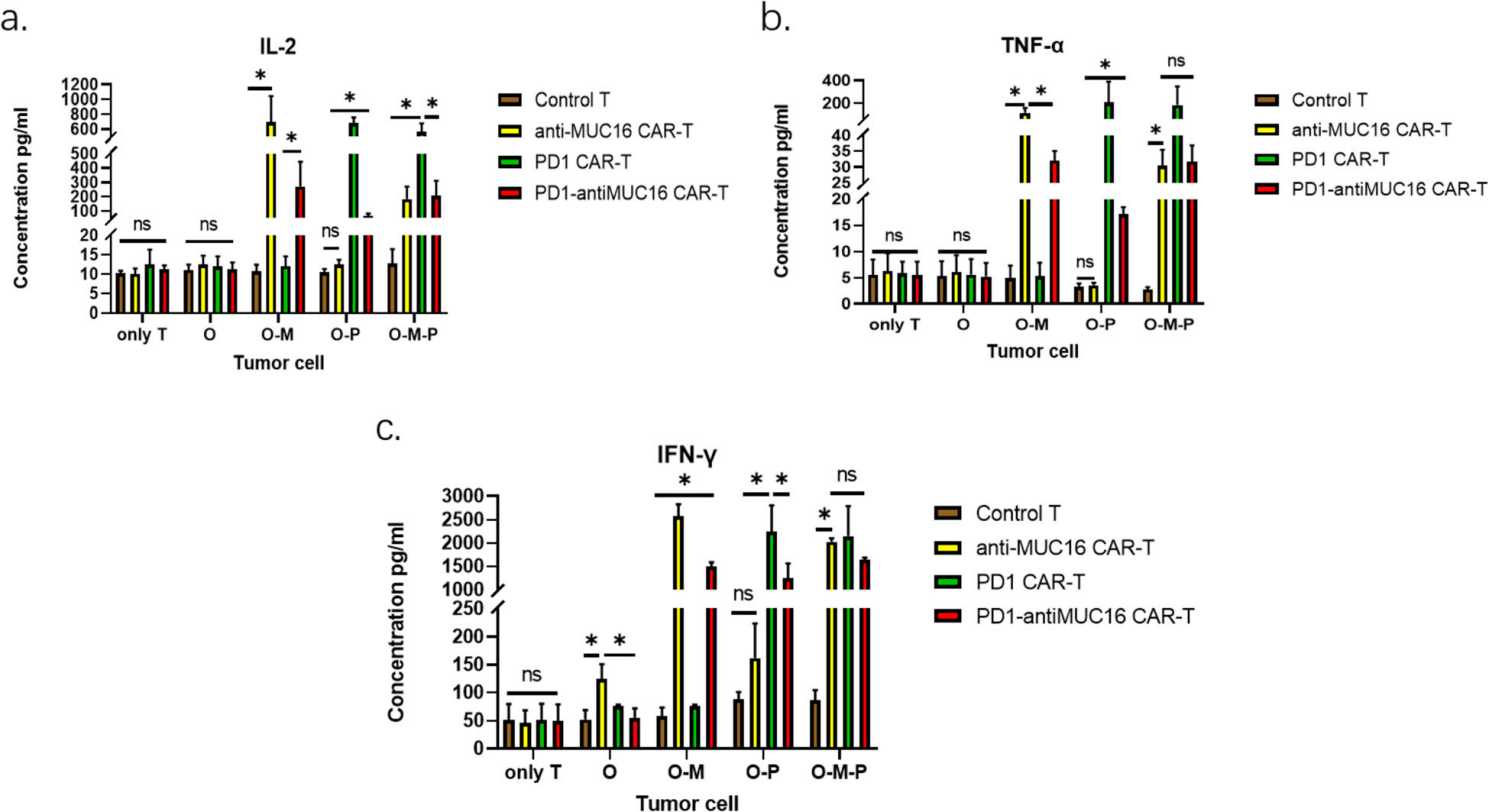
Three cytokines production by effector cells in response to various OVCAR-3 cell lines. Four effector cells (PD1-antiMUC16 CAR-T cells, PD1 CAR-T cells, anti-MUC16 CAR-T cells and T cells without CAR molecule) co-cultured with four kinds of OVCAR-3 cells (OVCAR3-luc cells, OVCAR3-MUC16-GFP-luc cells, OVCAR3-PDL1-luc cells, and OVCAR3-MUC16-GFP-PDL1-luc cells) at 1:1 ratio for 48 h to explore the different secretion capacities of IL-2, IFN-γ, and TNF-α between four T cells. These cytokines tested by ELISA kits. Only T: without target cells, O: OVCAR3-luc cell, O-M: OVCAR3-MUC16-GFP-luc cell, O-P: OVCAR3-PDL1-luc cell, O-M-P: OVCAR3-MUC16-GFP-PDL1-luc cell, −:The groups covered by horizontal line are compared in pairs. Data show the mean ± SD, and the results were analyzed with the nonparametric test. Error bars represent the SD.ns: *P* > 0.05, *: *p* < 0.05

In cytokine release test to assess whether CAR structure enhanced the anti-tumor activity of T cells, co-cultures were established between CAR-T cells and target cells at 1:1 ratio (1 × 10^4^ T cells versus 1 × 10^4^ cancer cells) in a V-bottomed 96-well plate for 48 h in incubator. The results revealed all CAR-T cells exerted a more robust capacity of secreting IL-2, IFN-γ, and TNF-α (Fig. 6) which were harvested from the supernatants and measured by ELISA kits. Disappointingly, dual CAR-T cells did not reveal higher levels of cytokines production than single CAR-T cells.

### Functional activity of CAR-T cells in vivo

**Fig. 7:**
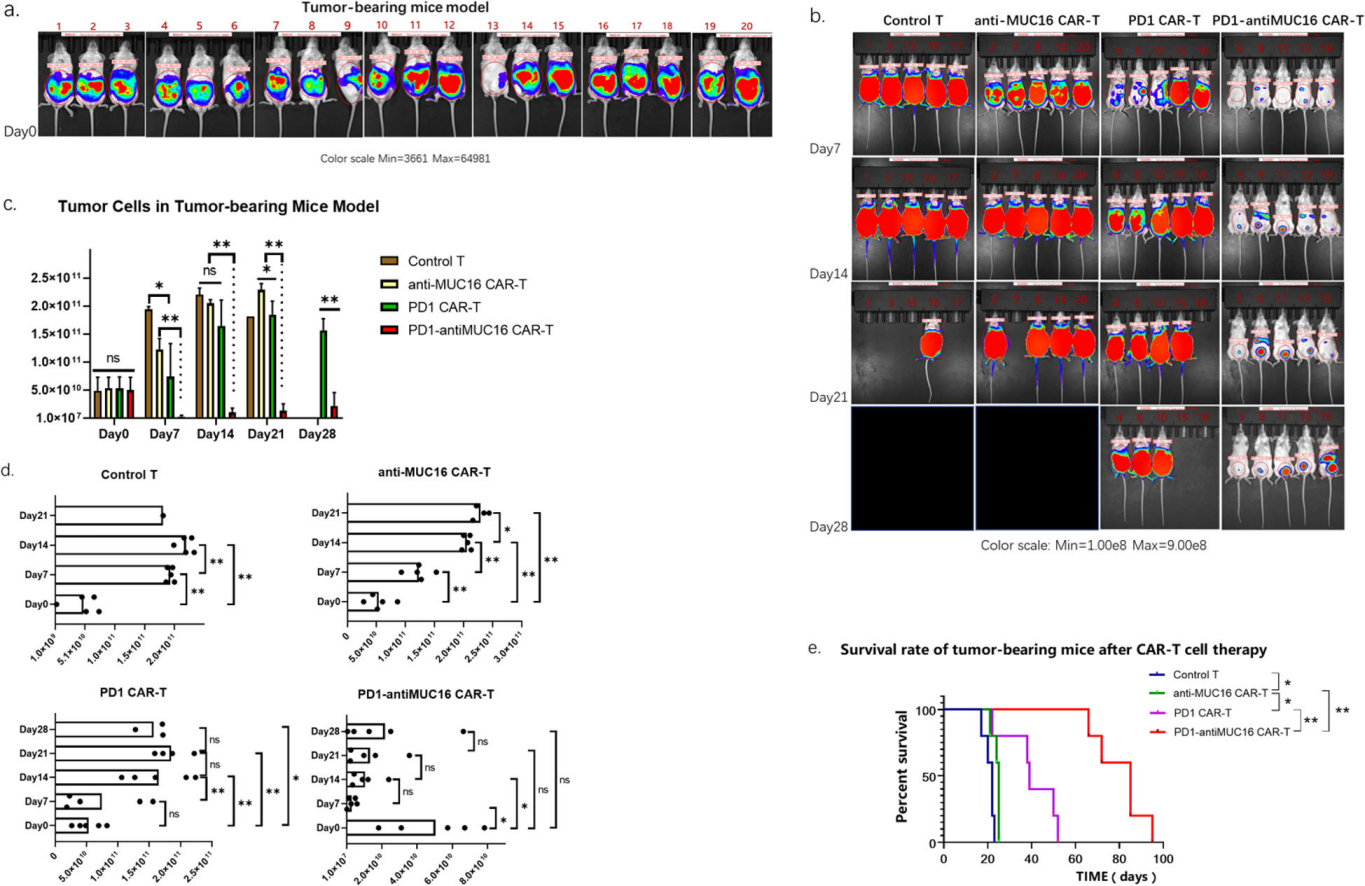
Therapeutic efficacy of CAR-T cells on the xenograft mice model. **a** The Vivo imaging results of OVCAR3-MUC16-GFP-PDL1-luc tumor-bearing mice before treatment. NPG mice were intraperitoneally injected with OVCAR3-MUC16-GFP-PDL1-luc cells (5 × 10^5^) and 50ul Matrigel for 48 h, followed by measuring via IVIS Spectrum and analyzed by Living Image. **b** The Vivo imaging results of tumor progression in mice at four points (day 7, day 14, day 21and day 28) in time, and were randomized divided into four groups. The black area and empty spaces indicate the mice have died. **c** The therapeutic effects of the four T cells are compared at the same point in time. **d** The therapeutic effect of each T cell group is compared at different points in time. **e** The survival curve of tumor-bearing mice. All groups below the horizontal line have been compared, the two groups pointed by the arrow line are compared. Data show the mean ± SD, and the results were analyzed with Student’t test. Error bars represent the SD. The survival curve was drawn by GraphPad Prism 8.3.0 software. ns: *P* > 0.05,*: *P* < 0.05,**: *P* < 0.01.

In order to determine the efficacy of CAR-T cells against ovarian cancer cells in vivo, we established intraperitoneal tumor-bearing models using NPG mice, which were injected with OVCAR3-MUC16-GFP-PDL1-luc cells (5 × 10^5^ cells) and 50 μL Matrigel into the abdominal cavity and raised 48 h. As shown in Fig. 7a, all mice appeared well-distributed with stable size tumors. They were measured for fluorescence values by IVIS Spectrum and analyzed for the pre-therapeutic evidence of tumor by Living Image.

Tumor-bearing mice models were randomized into four groups (*n* = 5 per group) and injected with CAR-T cells (1 × 10^6^ cells) into the abdominal cavity on day 0. The fluorescence values was then measured weekly to monitor the progress of the tumor. Dual CAR-T exhibited significant regression of ovarian cells as detected on day 7 to day 14 (Fig. 7b, c), followed by slow proliferation. However, two single CAR-T groups did not show the potent therapeutic effect as dual CAR-T cell did, with the progress of the tumor restrained instead. From all models, we discovered that the best treatment efficiency was shown for the first week after injecting CAR-T cells (Fig. 7d). This may help establish the dose and frequency of future safety and clinical trials.

As time passed, all four groups of mice died for ovarian cancer; however, their tumor-bearing survival time was different. The dual CAR-T group demonstrated exceptionally longer survival time of mice than the single CAR-T groups and control group. The mean survival time of dual CAR-T group reached to 80.6 ± 10.33 days. Whereas for two single CAR-T groups, the mean survival times of PD1 CAR-T group and antiMUC16 CAR-T group were 45.2 ± 6.34 days and 23.0 ± 1.55 days, respectively. The control group had the shortest survival time of 19.8 ± 2.14 days (Fig. 7e). From the perspective of extending the lifetime of tumor-bearing mice, the dual CAR-T group demonstrated the higher capacity of prolonging the survival time of mice than others (*P* < 0.01) (Fig. 7e).

## Discussion

The aggressive ovarian cancer as the exceptionally high-grade serous carcinoma is deemed as an urgent medical challenge in the twenty-first century due to the low five-year survival time, rapidly invasive progression and high recurrence rate. CAR-T technology has exhibited feasible antitumor activities in hematologic malignancies with a potential in the treatment of OC. However, the paucity of specific antigens and the immune escape of OC are the primary obstacle.

In our study, in order to increase the target specificity and reduce immune escape, we adopted a tandem structure in design of CAR molecule for two antigenic targets using second-generation CAR-T conception. The results indicated that both two CARs showed antitumor activity rather than interacting with each other, which may be attributed to the hinge domain supplying space for scFv folding [29–31]. MUC16 and PDL1 are undoubtedly ideal target antigens for CAR-T technology against OC in our study. Additionally, both dual CAR-T cells and single CAR-T cells showed favorable cytotoxic efficiency against various devised OVCAR-3 cells in vitro, especially at a high E/T ratio. The destructive effect of dual CAR-T cells is not superior to that of the single CAR-T cells. Although there is no apparent difference between the dual CAR-T cell and single CAR-T cell in cytotoxicity and cytokines production in vitro, dual CAR-T demonstrated remarkable tumor therapeutic effect in vivo and prolonged survival time of tumor-bearing mice models as compared with that of single CAR-T cells.

To understand why dual CAR-T cells exhibited disparity tumor therapeutic effects for in vivo and in vitro, the critical point to consider may be the fact that PD1 recognizes target antigens, which are correlated with the tumor microenvironment. This environment which is not developed in vitro may have facilitated the high therapeutic effect in vivo. This assumption was supported by the assay results. PD1 CAR-T exhibited potent cytotoxicity in mice models and significantly prolonged the survival, especially against OVCAR3-PDL1-luc cells and OVCAR3-MUC16-GFP-PDL1-luc cells. The assay results indicated a significant attack capability of CAR-T cells against target cells impacted by the surrounding environment. In the experimental design, PD1-antiMUC16 CAR-T were constructed as the experimental group, while PD1 CAR-T and anti-MUC16 CAR-T were used as the positive control, and control T without any CAR structure was used as the negative control. Additionally, PDL1 antigen was induced in up-regulation by activated T cells in vivo rather than high natural expression. We established a variety of tumor cells, i.e., OVCAR-3 cells, OVCAR3-PDL1-luc cell, OVCAR3-MUC16-GFP-luc cell, and OVCAR3-PDL1-MUC16-GFP-luc cells to better simulate antigenic expression in OC patients. To build a desirable model that will exclude the impact of different antigen expression rates in patients and simulate the condition in vivo, we constructed the OVCAR3-PDL1-MUC16-GFP-luc tumor-bearing mice model. It is also worth noting that immunosuppressive tumor microenvironment existing in tumor-bearing mice nay partially imitate the human condition but still quite different. We acknowledge further animal studies are needed to demonstrate the advantage of CAR-T technology.

Another factor that may impact the activity of CAR-T cells is the spatial conformation of extracellular domains. Leonard Leong et al. verified that the structure of the part behind the antigen-recognize region and its length could affect the activity of CAR-T cells [32]. Based on this finding, the cytotoxicity of CAR-T cells may be related to the composition and spatial conformation of extracellular domains. The prospect to optimize the CAR molecules by exploring the spatial conformation of extracellular domains appears promising.

There are indeed knowledge gaps in research, such as how to reduce the CRS, improve homing, and keep consistency in OC patients. Previously, some researchers had proposed that selecting target antigens with high specificity, optimizing the function of CAR-T cells, and blocking immunosuppressive molecules such as PD1/PDL1 signal, could reduce the occurrence of CRS [33–37]. Moreover, combining higher specific CAR-T cells with chemokine receptors may improve homing and enhance therapeutic activity [6]. On the other hand, some researchers believe that CAR-T does not lead to CRS and other side effects. Wen H et al. verified that CAR-T19 had no significant immunotoxicity, including the mean body mass, blood cells counts, and no CRS was observed by detecting IL-10, IL-6, IFN-γ, and TNF in adult acute lymphoblastic leukemia NSG mice models [38]. However, the study of CAR-T side effects is not involved in this study, which need to be studied by preclinical trials. For future studies of dual CAR-T cells against EOC, we plan to work on topics such as optimal injection dose, treatment cycle, and further preclinical safety evaluation.

## Conclusion

We demonstrated that PD1-antiMUC16 dual-target, and single-target CAR-T cells possess cytotoxicity against OVCAR-3 cell line expressing PDL1 and MUC16 antigens and induce cytokines release in vitro. Dual CAR-T cells demonstrated advantages of therapeutic effect on OVCAR3-MUC16-GFP-PDL1-luc tumor-bearing mice and significantly prolonged survival time. Single CAR-T cells also inhibited tumor cell proliferation in tumor-bearing models and prolonged the survival time. PD1-antiMUC16 CAR-T cells demonstrated a therapeutic effect and the experimental data may support further research work that will potentially lead to clinical studies.

## Supplementary information

**Additional file 1: Figure S1.** Full-length gels of dual-target CAR. a, 1 KB marker was used as a standard marker. Anti-MUC16 F fragment and anti-MUC16 R fragment were utilized for constructing the anti-MUC16 fragment. PD1-M was performed as a mock form of PD1. The left side of the clipping line was Fig. 2. The base length of anti-MUC16 F, anti-MUC16 R, PD1-M, and PD1 was 813 bp,510 bp,700 bp, and 510 bp, respectively. b, All bands were anti-MUC16 fragments and the length about 1500 bp. The two bands on the left, more evident than the others, were displayed for subsequent experiments. c, The 8000 bp band and 10,000 bp band in standard bands were not wholly distinguished. Mock marked a 7000 bp band. PLV-PD1-antiMUC16 plasmid consisted of a dual CAR structure with a 2000 bp band and a base skeleton with 7435 bp. d, After amplifying in bacterial solution, PD1-antiMUC16 was measured by agarose gel electrophoresis. Mock marked 1500 bp band. All images of gel were performed by DNA sequence analysis of electrophoresis apparatus (LIUYI BIOTECHNOLOGY, Beijing, China).

## Data Availability

Data supporting the results in the article are available from the corresponding author upon reasonable request.

## Abbreviations

EOC: Epithelial ovarian cancer
OC: Ovarian cancer
CAR-T: Chimeric antigen receptor T
IL-2: Interleukin-2
IFN-γ: Interferon-γ
TNF-α: Tumor necrosis factor-α
CRS: Cytokine release syndrome
MUC16: Mucin 16
PD1: Programmed cell death-1
PDL1: Programmed cell death-ligand 1
CTLA-4: Cytotoxic T lymphocyte-associated antigen-4
UBMC: Umbilical blood mononuclear cell
FBS: Fetal bovine serum
scFv: Single chain antibody fragment
FDA: Food and Drug Administration
PBS: Phosphate buffer saline
PEI: Polyetherimide
GFP: Green fluorescent protein
SPF: Specific pathogen-free
