# Supplementary figures and images for: Therapeutic effect of dual CAR-T targeting PDL1 and MUC16 antigens on ovarian cancer cells in mice
Source: BMC Cancer. 2020 Jul 20;20:678. doi: 10.1186/s12885-020-07180-x (PMC7372885; doi:10.1186/s12885-020-07180-x)

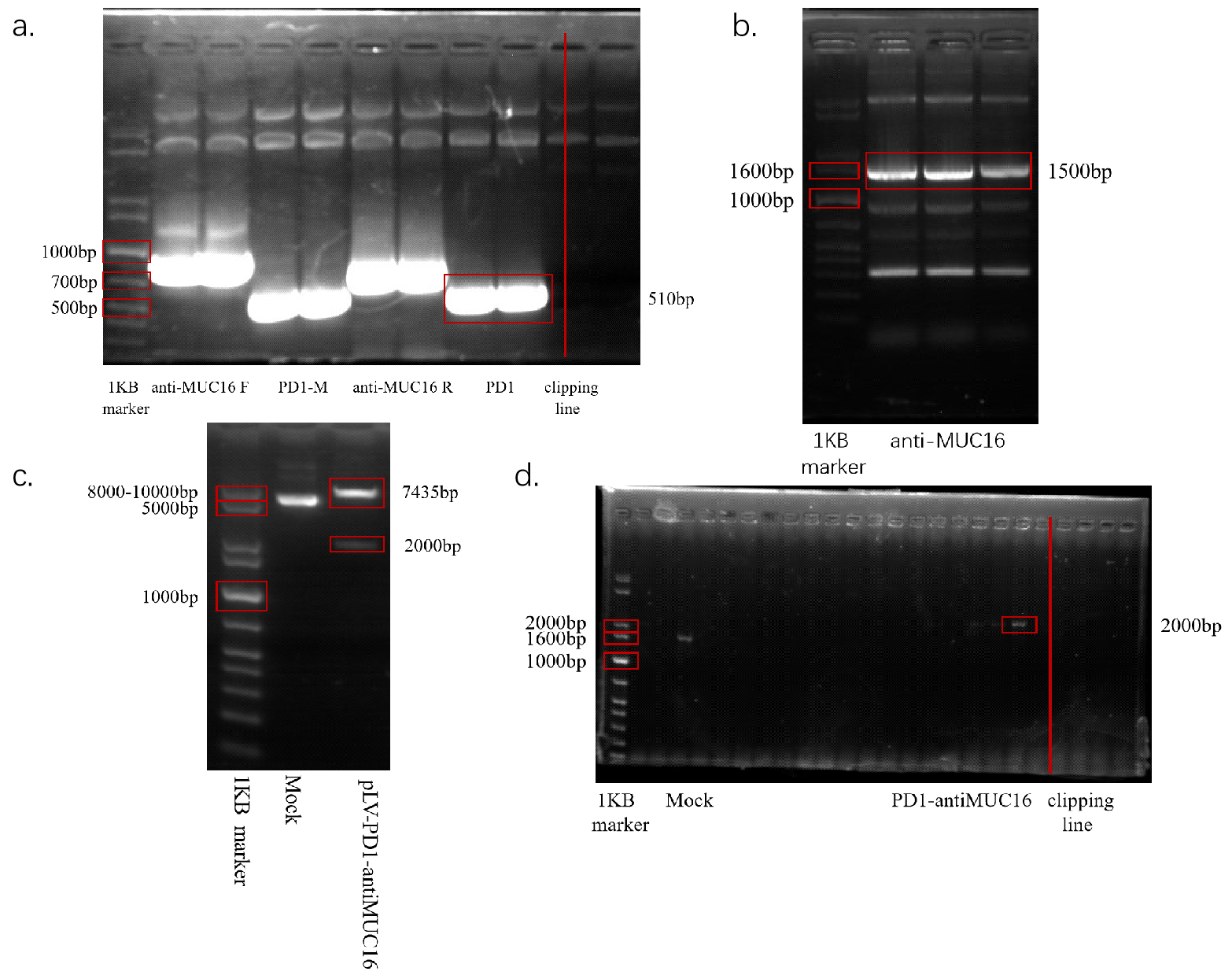

Supplement: Supplementary file 1 — Additional file 1: Figure S1. Full-length gels of dual-target CAR. a, 1 KB marker was used as a standard marker. Anti-MUC16 F fragment and anti-MUC16 R fragment were utilized for constructing the anti-MUC16 fragment. PD1-M was performed as a mock form of PD1. The left side of the clipping line was Fig. 2. The base length of anti-MUC16 F, anti-MUC16 R, PD1-M, and PD1 was 813 bp,510 bp,700 bp, and 510 bp, respectively. b, All bands were anti-MUC16 fragments and the length about 1500 bp. The two bands on the left, more evident than the others, were displayed for subsequent experiments. c, The 8000 bp band and 10,000 bp band in standard bands were not wholly distinguished. Mock marked a 7000 bp band. PLV-PD1-antiMUC16 plasmid consisted of a dual CAR structure with a 2000 bp band and a base skeleton with 7435 bp. d, After amplifying in bacterial solution, PD1-antiMUC16 was measured by agarose gel electrophoresis. Mock marked 1500 bp band. All images of gel were performed by DNA sequence analysis of electrophoresis apparatus (LIUYI BIOTECHNOLOGY, Beijing, China). [file 12885_2020_7180_MOESM1_ESM.tif]
